# Evaluation of Mortality Risk Factors in Prostate Cancer: Impact of Demographic, Clinical, Laboratory, Therapeutic, and Trace Element Influences

**DOI:** 10.1002/cnr2.70166

**Published:** 2025-03-11

**Authors:** Pitchou Mukaz Mbey, Pablo Diasiama Kuntima Diangienda, Olivier Mukuku, Willy Kalau Arung, Célestin Lubaba Banza, Alpha Tsita Mafuta, Mathieu Nkumu Loposso, Bienvenu Massamba Lebwaze, Augustin Maole‐Lembe Punga, Guy Ilunga Nday, Dieudonné Molamba Moningo

**Affiliations:** ^1^ Department of Surgery, Faculty of Medicine University of Lubumbashi Lubumbashi Democratic Republic of the Congo; ^2^ Department of Surgery, Urology, Faculty of Medicine University of Kinshasa Kinshasa Democratic Republic of the Congo; ^3^ Institut Supérieur Des Techniques Médicales Lubumbashi Democratic Republic of the Congo; ^4^ Department of Toxicology, Faculty of Medicine University of Lubumbashi Lubumbashi Democratic Republic of the Congo; ^5^ Department of Histopathology, Faculty of Medicine University of Kinshasa Kinshasa Democratic Republic of the Congo

**Keywords:** mortality, prostate cancer, risk factors, trace elements

## Abstract

**Background:**

Prostate cancer (PCa) is a significant contributor to male mortality globally, including in the Democratic Republic of the Congo (DRC). Various factors play a role in its onset and progression. The impact of trace elements and other risk factors on the survival of PCa patients is not extensively studied in this setting. This study aimed to evaluate the effect of demographic characteristics, clinical factors, laboratory investigations, therapeutic aspects, and trace elements on the occurrence of mortality in this disease.

**Methods:**

A sample of 94 PCa patients was included in this study. Among them, 22 (23.40%) deceased, while 72 (76.60%) survived during a 5‐year follow‐up period. Sociodemographic, clinical, laboratory investigations, therapeutic aspects, and trace element levels (in tissues and urine) were gathered and analyzed. Statistical analyses were conducted to pinpoint mortality predictors, with Cox regression utilized to account for variable impacts.

**Results:**

In multivariate analyses, age (adjusted hazard ratio [aHR] = 1.06; *p* = 0.025), prostate‐specific antigen [PSA] (aHR = 1.01; *p* = 0.037), hemoglobin (aHR = 0.69; *p* = 0.010), and the presence of metastases (aHR = 3.83; *p* = 0.037) were identified as significant predictors of mortality. Furthermore, elevated levels of urinary strontium (aHR = 1.08; *p* = 0.016), manganese (aHR = 1.51; *p* = 0.003), and cobalt (aHR = 1.30; *p* = 0.030) were linked to an increased risk of mortality. Conversely, higher levels of tissue copper were associated with a reduced risk of death (aHR = 0.99; *p* = 0.045).

**Conclusion:**

The results obtained have indicated that specific trace elements, along with age, PSA level, hemoglobin level, and the presence of metastases, are predictive of mortality in PCa patients in the DRC. Enhanced comprehension and control of these factors may lead to improved survival outcomes. Additional investigation is warranted to validate these correlations and to facilitate the development of therapeutic interventions.

## 1 Introduction

Prostate cancer (PCa) remains a critical public health problem, consistently ranking as the second most frequently diagnosed cancer in men worldwide [1, 2]. The prevalence and mortality of this disease vary considerably from region to region and population to population, with particularly alarming trends observed in regions where access to healthcare and cancer screening programs is limited [3]. The Democratic Republic of the Congo (DRC) is one such region where PCa mortality remains a major concern, reflecting the challenges faced by low‐income countries in managing chronic disease. According to recent estimates based on all cancers recorded in the DRC, PCa is second only to breast cancer in terms of frequency and mortality: the incidence rate has been estimated at 14.8%, prevalence over a 5‐year period at 12.6 per 100 000 population, and mortality at 12.8% [2]. Despite numerous efforts to improve PCa outcomes, the prognosis remains poor. It remains one of the leading causes of cancer‐related mortality, with the highest death rate nationally. Although individual screening programs enable early diagnosis and a subsequent reduction in mortality rates, their routine availability remains challenging [4]. In some parts of the world, PCa is often diagnosed at an advanced stage, severely limiting treatment options and leading to poor five‐year survival rates [3]. This is the case in the DRC, where hospital statistics show that 47.1% of PCa cases are diagnosed at the metastatic stage, with a median survival of 30 months and an average survival of 26.6 months [5].

The clinical stage of the disease is one of the most crucial but non‐modifiable factors influencing prognosis. Therefore, the identification and accessibility of prognostic markers beyond the stage are becoming imperative for more accurate detection of cancer progression and the implementation of personalized treatment strategies [6, 7, 8, 9]. Trace elements have been shown to potentially play a role in the development, progression, and response to treatment of PCa [10]. There is an increased demand for copper in proliferating cancer cells compared to other tissues, suggesting a potential metabolic vulnerability that could be exploited by controlling copper availability during cancer progression. The concentrations of trace elements may play a crucial role in cancer development by influencing proliferation or apoptosis. Disruption of the balance between free radicals and antioxidants can cause cell damage and trigger carcinogenesis [11, 12]. Despite studies exploring the prediction of mortality based on sociodemographic, clinical, and laboratory investigations in PCa [3, 13, 14], although trace elements are known to influence cancer progression, few studies have investigated their impact on mortality in PCa patients [15, 16, 17]. This study seeks to fill that gap by evaluating how demographic, clinical, laboratory, therapeutic factors, and trace elements contribute to mortality in PCa patients in this setting. The role of trace elements, including cobalt, copper, zinc, and selenium, has been highlighted in previous research [15, 16, 17], but further exploration in African populations is necessary.

This study aims to further evaluate the risk factors for mortality in PCa within a different environmental context and to assess the effect of demographic characteristics, clinical factors, laboratory investigations, therapeutic aspects, and trace elements on the occurrence of mortality in this disease. We aim to identify these risk factors to inform tailored treatment strategies that can improve patient outcomes in this population.

## 2 Materials and Methods

### 2.1 Setting, Type, and Period of Study

This longitudinal analytical study was conducted among PCa patients residing in Haut‐Katanga province, in the Democratic Republic of the Congo, from April 5, 2015, to April 5, 2023. Exhaustive sampling included all patients followed up for PCa at Cliniques Universitaires de Lubumbashi, Centre Arc‐en‐ciel Baraka, Med Park Clinic, and Polyclinique CMDC, all located in Lubumbashi.

### 2.2 Study Population

**FIGURE 1:**
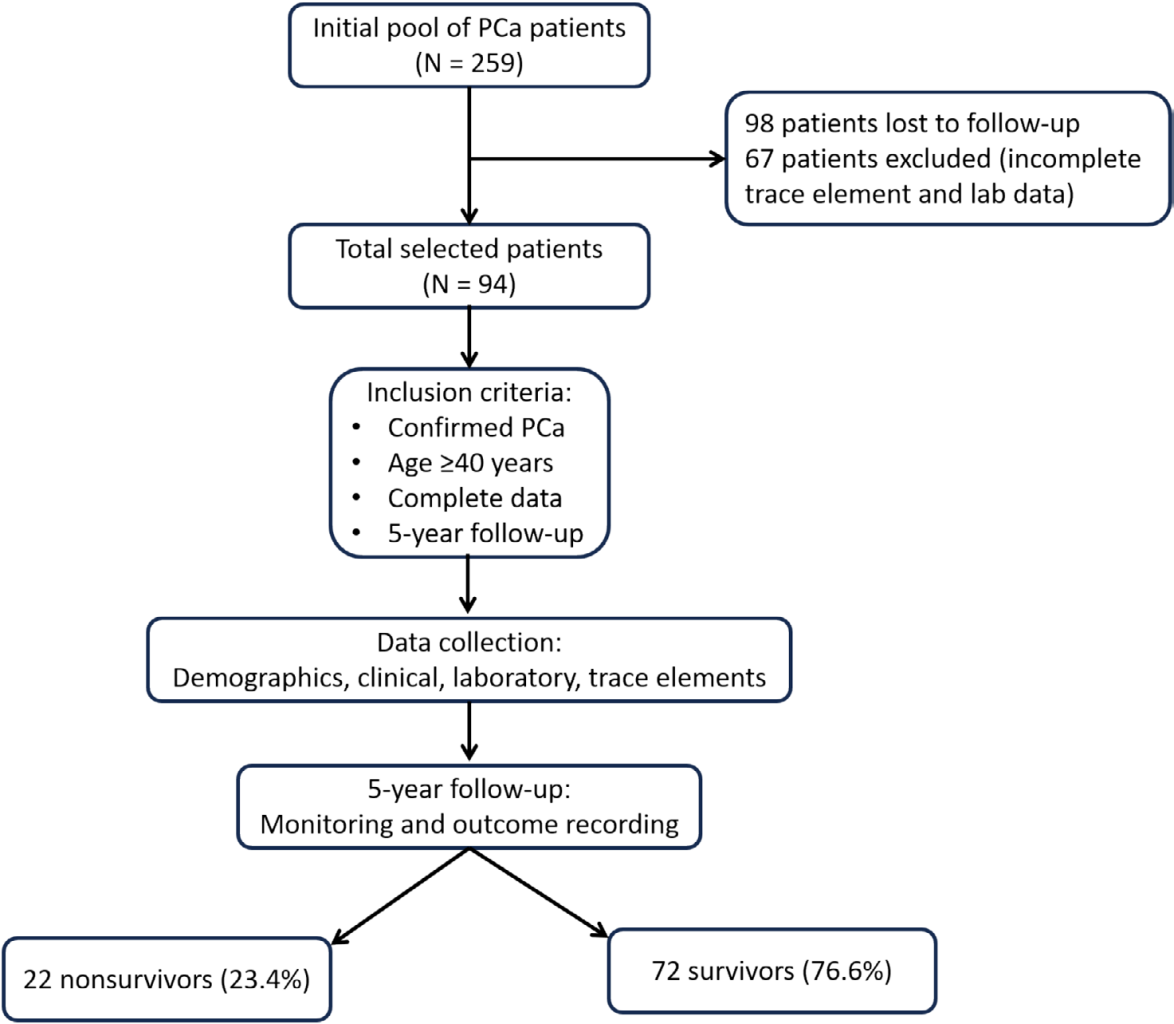
Flowchart for patient selection.

The study population consisted of adult men undergoing follow‐up for PCa. Inclusion criteria required documented PCa confirmed by a twelve‐core biopsy obtained under ultrasound guidance and stored at −20°C in the laboratory of Cliniques Universitaires de Lubumbashi, as well as a 24‐h or waking urine sample preserved in EDTA‐containing vials for trace element analysis. A comprehensive medical record was necessary for inclusion in the study, resulting in a sample size of 94 PCa patients (Figure 1).

### 2.3 Methods

Our variables of interest analyzed included the sociodemographic characteristics of the patients, such as age at diagnosis (< 60 years, 60–69 years, 70–79 years, or ≥ 80 years), level of education (primary, secondary, higher/university), and marital status (single, married, widowed, or divorced). In terms of clinical characteristics, we recorded the history of diabetes mellitus, arterial hypertension, digital rectal examination, post‐micturition residual, and the presence or absence of metastases. Therapeutic aspects were also documented according to the treatment protocol tailored to each patient's clinical stage and financial means. Treatments available in our setting included hormone therapy (Goserelin and Bicalutamide as first‐line, Abiraterone as second‐line), chemotherapy (Docetaxel), and surgery. Radiotherapy was not accessible in our setting. In laboratory investigations, we measured prostate‐specific antigen (PSA), serum creatinine, hemoglobin, and trace elements. Prostate volume was assessed using ultrasound. The definitive diagnosis of PCa was based on histopathological analysis, considering the Gleason score, the International Society of Urological Pathology (ISUP) grading, and d'Amico classification (low, intermediate, and high risk). Patients were monitored for 5 years, and their outcomes were categorized as death or survival.

The PSA assay was conducted utilizing a HumanReader HS and a PSA kit provided by Human Diagnostics Worldwide (65205 Wiesbaden, Germany). Prostate biopsies were carried out with disposable sterile needles (Tru‐Cut, 20 cm long and 20 mm in diameter) from Sterylab (Milan, Italy). The biopsy procedure was ultrasound‐guided, utilizing a digital color Doppler ultrasound system model S11 (Sonomed, 74‐00128 Rome, Italy) equipped with an endorectal probe with a biopsy needle attachment.

For each patient, 12 sextant biopsies were taken, with 2 samples collected from each site (left and right apex, left and right median, and left and right base). These samples were then fixed in 10% formalin in pre‐labelled vials, packaged, and subsequently sent for histological analysis.

The assays for trace elements in urine were conducted directly after the urine had thawed, while for prostate tissue, oven drying at 105°C was performed to eliminate moisture and solvents. The final dry tissue was weighed on a precision analytical balance, and the tissue digestion was carried out in a solution containing 30% concentrated hydrogen peroxide and 65% concentrated nitric acid. This process took place in a microwave oven gradually heated to a temperature of 250°C, with pressure ranging from 0 to 60 bar to prevent passing into the liquid phase before mineralization. The analysis was conducted at the provincial laboratory of the Office Congolais de Contrôle (OCC) in Lubumbashi, which has been ISO 9150 quality certified since 2010. The OptimaTM 8300 ICP‐OES Spectrometer from PerkinElmer in Waltham, USA, was used for the analysis. The calibration was done using a standard 3 solution, and the trace elements measured included Aluminum (Al), Arsenic (As), Cadmium (Cd), Cobalt (Co), Chromium (Cr), Copper (Cu), Magnesium (Mg), Manganese (Mn), Nickel (Ni), Lead (Pb), Selenium (Se), and Zinc (Zn).

### 2.4 Statistical Analysis

A database was created, and statistical analysis was conducted using STATA version 16 software. Descriptive analysis involved calculating proportions for qualitative variables (frequencies and percentages) and medians with interquartile ranges (IQR) for non‐normally distributed quantitative variables, following verification by the Shapiro–Wilk test. In this study, death served as the dependent variable. The Mann–Whitney *U* test or ANOVA test (where applicable) was utilized to compare medians between deceased and surviving patients.

Bivariate analysis was conducted to examine the relationship between the independent variables and death using Chi‐square (χ2). Following this, Cox regression was employed to calculate the adjusted hazard ratio (aHR) to determine risk factors for death in PCa patients. All clinically significant variables and those with a *p*‐value < 0.1 were incorporated into the regression model. A significance level of *p* < 0.05 was used for the multivariate analysis. The goodness of fit was assessed based on the criteria established by Hosmer and Lemeshow.

### 2.5 Ethical Considerations

Patients were informed about the study's purpose, and written informed consent was obtained before their inclusion. Approval was granted by the Ethics Committee of the University of Lubumbashi under approval number UNILU/CEM/015/2020.

## 3 Results

A total of 94 PCa patients were included in the present study, of whom 22 (23.40%) died and 72 (76.60%) survived after 5 years' follow‐up. The overall survival rate at 5 years was 76.60%. The median follow‐up time for non‐survivors was 16 months (IQR: 7 and 23 months).

Table 1 shows the sociodemographic and clinical characteristics of PCa patients (*n* = 94) stratified by clinical course. The median age was 69.50 years (IQR: 64.00–75.00); non‐survivors were significantly older than survivors (73.50 years vs. 67.50 years; *p* = 0.0011). During clinical examination, rectal touch was pathological in 90.91% of non‐survivors and 66.67% of survivors; a statistically significant difference was observed between the two groups (*p* = 0.0260). Metastases were present in 16.67% of surviving patients and 54.55% of non‐survivors, with a highly significant difference noted in the statistical analysis (*p* < 0.0001). No significant associations were found between mortality and variables such as education level, marital status, history of diabetes mellitus, history of hypertension, body mass index, and TNM classification (*p* > 0.05).

**TABLE 1:** Socio‐demographic and clinical characteristics of prostate cancer patients (*N* = 94) stratified by clinical course.

Variable	Total (*N* = 94), *n (%)*	Non‐survivors (*n* = 22), *n (%)*	Survivors (*n* = 72), *n (%)*	*p* value
Age				0.0104
< 60 years	9 (9.57)	1 (4.55)	8 (11.11)	
60–69 years	38 (40.43)	4 (18.18)	34 (47.22)	
70–79 years	36 (38.30)	11 (50.00)	25 (34.72)	
≥ 80 years	11 (11.70)	6 (27.27)	5 (6.94)	
Median (IQR)	69.50 (64.00–75.00)	73.50 (71.00–80.00)	67.50 (63.00–72.00)	0.0011
Level of education				0.7395
Primary	14 (14.89)	4 (18.18)	10 (13.89)	
Secondary	35 (37.23)	9 (40.91)	26 (36.11)	
Higher/University	45 (47.87)	9 (40.91)	36 (50.00)	
Marital status				0.4373
Single	4 (4.26)	2 (9.09)	2 (2.78)	
Married	76 (80.85)	17 (77.27)	59 (81.94)	
Widower	14 (14.89)	3 (13.64)	11 (15.28)	
Diabetes mellitus				0.3844
No	82 (87.23)	18 (81.82)	64 (88.89)	
Yes	12 (12.77)	4 (18.18)	8 (11.11)	
Hypertension				0.2226
No	73 (77.66)	15 (68.18)	58 (80.56)	
Yes	21 (22.34)	7 (31.82)	14 (19.44)	
Body mass index (kg/m^2^)				0.5769
18.5–24.9	65 (69.15)	15 (68.18)	50 (69.44)	
25.0–29.9	26 (27.66)	7 (31.82)	19 (26.39)	
≥ 30.0	3 (3.19)	0 (0.00)	3 (4.17)	
Median (IQR)	24.00 (23.00–26.00)	24.00 (23.00–26.00)	24.00 (22.50–26.00)	0.9354
Rectal touch				0.0260
Normal	26 (27.66)	2 (9.09)	24 (33.33)	
Pathological	68 (72.34)	20 (90.91)	48 (66.67)	
Metastasis				< 0.0001
Absent	70 (74.47)	10 (45.45)	60 (83.33)	
Present	24 (25.53)	12 (54.55)	12 (16.67)	
TNM classification				0.5411
cT1	35 (37.23)	8 (36.36)	27 (37.50)	
cT2	20 (21.28)	3 (13.64)	17 (23.61)	
cT3	28 (29.79)	9 (40.91)	19 (26.39)	
cT4	11 (11.70)	2 (9.09)	9 (12.50)	

The laboratory investigations of PCa patients (*N* = 94) stratified by clinical course are outlined in Table 2. The data demonstrate notable distinctions between non‐survivors and survivors across various clinical parameters. Non‐survivors exhibited a higher median PSA level (124.56 ng/mL) in comparison to survivors (86.25 ng/mL), elevated serum creatinine levels (1.20 mg/dL vs. 0.90 mg/dL), lower median hemoglobin levels (9.85 g/dL vs. 11.00 g/dL), and a higher Gleason score (8 vs. 7). These findings suggest that elevated PSA levels, compromised renal function, anemia, and increased tumor aggressiveness indicated by the Gleason score are correlated with mortality in PCa patients.

**TABLE 2:** Laboratory investigations and therapeutic aspects of prostate cancer patients (*N* = 94) stratified by clinical course.

Variable	Total (*N* = 94), *n (%)*	Non‐survivors (*n* = 22), *n (%)*	Survivors (*n* = 72), *n (%)*	*p* value
PSA (ng/mL)				0.4527
≤ 20	18 (19.15)	3 (13.64)	15 (20.83)	
> 20	76 (80.85)	19 (86.36)	57 (79.17)	
Median (IQR)	93.04 (45.80–162.20)	124.56 (72.00–312.00)	86.25 (39.61–156.27)	0.0307
Serum creatinine (mg/dL)				0.5339
0–1.2	64 (68.09)	13 (59.09)	51 (70.83)	
1.3–2.0	15 (15.96)	5 (22.73)	10 (13.89)	
> 2.0	15 (15.96)	4 (18.18)	11 (15.28)	
Median (IQR)	0.93 (0.77–1.90)	1.20 (1.00–2.00)	0.90 (0.70–1.90)	0.0032
Hemoglobin (g/dL)				0.0074
< 10	26 (27.66)	11 (50.00)	15 (20.83)	
≥ 10	68 (72.34)	11 (50.00)	57 (79.17)	
Median (IQR)	11.00 (9.30–12.00)	9.85 (8.00–11.00)	11.00 (10.00–12.00)	0.0032
Prostate volume (mL)				0.8534
≤ 60	5 (5.32)	1 (4.55)	4 (5.56)	
> 60	89 (94.68)	21 (95.45)	68 (94.44)	
Median (IQR)	85.50 (73.00–98.00)	85.00 (77.00–138.00)	85.50 (72.50–97.00)	0.4110
Post‐micturition residue				0.2937
< 50 mL	40 (42.55)	7 (31.82)	33 (45.83)	
50–100 mL	23 (24.47)	8 (36.36)	15 (20.83)	
> 100 mL	31 (32.98)	7 (31.82)	24 (33.33)	
Median (IQR)	60.50 (35.00–210.00)	77.00 (38.00–570.00)	58.00 (32.00–158.50)	0.3912
Gleason score				0.0949
< 7	15 (15.96)	1 (4.55)	14 (19.44)	
≥ 7	79 (84.04)	21 (95.45)	58 (80.56)	
Median (IQR)	60.50 (35.00–210.00)	8.00 (7.00–8.00)	7.00 (7.00–8.00)	0.0173
d'Amico score				0.5127
Low	16 (17.02)	2 (9.09)	14 (19.44)	
Intermediate	13 (13.83)	3 (13.64)	10 (13.89)	
High risk	65 (69.15)	17 (77.27)	48 (66.67)	
IPSS score				0.1041
Light	36 (38.30)	6 (27.27)	30 (41.67)	
Moderate	21 (22.34)	3 (13.64)	18 (25.00)	
Severe	37 (39.36)	13 (59.09)	24 (33.33)	
ISUP 2016				0.0593
G1	15 (15.96)	1 (4.55)	14 (19.44)	
G2	11 (11.70)	2 (9.09)	9 (12.05)	
G3	30 (31.91)	5 (22.73)	25 (34.72)	
G4	31 (32.98)	10 (45.45)	21 (29.17)	
G5	7 (7.45)	4 (18.18)	3 (4.17)	
Treatment				0.3579
Hormone therapy	68 (72.34)	14 (63.64)	54 (75.00)	
Hormone therapy + pulpectomy	11 (11.70)	5 (22.73)	6 (8.33)	
Hormone therapy + chemotherapy	9 (9.57)	2 (9.09)	7 (9.72)	
Hormone therapy + chemotherapy + pulpectomy	3 (3.19)	1 (4.55)	2 (2.78)	
Hormone therapy + radical prostatectomy	3 (3.19)	0 (0.00)	3 (4.17)	

After Cox regression (Table 3), age was found to be a determinant of 5‐year mortality. For each increase of one unit in the age of a PCa patient, the risk of mortality increased by 6% on average (adjusted Hazard Ratio = 1.06; 95% CI: 1.01–1.11; *p* = 0.025). Similarly, PSA appears to be a significant predictor of mortality in PCa patients, although the effect is small. For each increase of one unit in PSA, the risk of mortality increases by 1% (adjusted Hazard Ratio = 1.01; 95% CI: 1.00–1.05; *p* = 0.037). Hemoglobin was also found to be significantly associated with mortality in PCa patients. For each increase of one unit in hemoglobin levels, the risk of mortality fell by 31% (adjusted Hazard Ratio = 0.69; 95% CI: 0.52–0.92; *p* = 0.010). The presence of metastases was strongly associated with an increased risk of mortality in PCa patients (adjusted Hazard Ratio = 3.83; 95% CI: 1.09–13.51; *p* = 0.037). The risk of mortality was almost quadrupled for patients with metastases compared with those without.

**TABLE 3:** Cox regression between mortality and patient characteristics in 94 prostate cancer patients.

Variable	aHR	SE	*t*‐value	*p* value	95% CI
Age	1.06	0.03	2.24	0.025	1.01–1.11
Prostate‐specific antigen	1.01	0.00	2.09	0.037	1.00–1.05
Serum creatinine	0.66	0.25	−1.13	0.259	0.32–1.36
Haemoglobin	0.69	0.10	−2.56	0.010	0.52–0.92
Metastasis present	3.83	2.46	2.09	0.037	1.09–13.51
Gleason score	0.71	0.21	−1.17	0.243	0.41–1.26

Abbreviations: 95% CI, 95% confidence interval; aHR, adjusted hazard ratio; SE, standard error.

Table 4 shows the median concentrations of trace elements in the prostate tissue and urine of 94 PCa patients divided according to their clinical course.

**TABLE 4:** Trace elements concentrations in prostate tissue (μg/dL) and urine (μg/g creatinine) in 94 prostate cancer patients.

Trace element	Total (*N* = 94), *Median (IQR)*	Non‐survivors (*n* = 22), *Median (IQR)*	Survivors (*n* = 72), *Median (IQR)*	*p* value^a^
Urine				
Arsenic	6.65 (4.10–13.10)	6.70 (4.30–14.80)	6.55 (4.05–13.00)	0.6714
Cadmium	0.10 (0.00–0.30)	0.10 (0.00–0.40)	0.10 (0.00–0.30)	0.5604
Cobalt	1.20 (0.90–1.60)	1.55 (1.20–1.80)	1.10 (0.80–1.45)	0.0022
Chromium	0.10 (0.00–0.80)	0.32 (0.00–1.20)	0.10 (0.00–0.75)	0.7900
Copper	18.20 (10.40–49.00)	29.75 (12.20–112.60)	18.00 (10.20–42.10)	0.1954
Manganese	0.01 (0.00–0.20)	0.02 (0.00–0.40)	0.01 (0.00–0.15)	0.0654
Lead	0.01 (0.00–0.50)	0.05 (0.00–0.90)	0.01 (0.00–0.50)	0.2344
Selenium	22.40 (12.70–31.20)	21.60 (13.20–31.00)	23.60 (12.50–34.10)	0.8829
Strontium	4.80 (1.30–13.20)	11.40 (1.90–31.00)	3.30 (1.10–8.65)	0.0175
Zinc	74.10 (28.90–187.90)	142.15 (42.80–254.60)	69.35 (27.85–180.70)	0.3216
Prostate tissue				
Aluminium	89.35 (46.00–143.00)	88.50 (28.80–118.10)	89.95 (46.15–146.05)	0.4372
Arsenic	1.15 (0.00–2.80)	1.05 (0.00–3.40)	1.15 (0.00–2.70)	0.9783
Cobalt	2.10 (1.60–3.90)	2.65 (1.60–4.10)	2.05 (1.65–3.70)	0.4605
Chromium	38.15 (15.70–65.90)	35.80 (10.80–71.90)	39.65 (16.70–63.55)	0.3718
Copper	124.50 (69.10–193.70)	105.65 (93.60–145.80)	129.70 (69.05–195.50)	0.5767
Manganese	5.10 (2.50–7.90)	6.05 (1.60–8.70)	5.00 (2.50–7.90)	0.6551
Nickel	24.85 (6.50–44.90)	25.75 (3.60–46.60)	24.85 (7.60–41.05)	0.8095
Lead	1.10 (0.10–2.70)	1.80 (0.20–3.90)	0.90 (0.05–1.90)	0.3049
Selenium	7.20 (5.20–9.90)	8.00 (6.20–14.30)	6.95 (4.90–9.80)	0.2000
Strontium	0.20 (0.10–0.20)	0.20 (0.10–0.20)	0.20 (0.10–0.20)	0.5922
Zinc	17.75 (12.40–22.10)	19.80 (14.60–27.70)	17.65 (12.05–21.50)	0.2920

^a^ Mann–Whitney test.

Table 5 displays correlations between trace element levels and the mortality risk in PCa patients. We identified a positive significant relationship between the concentrations of urinary strontium (aHR = 1.08; 95% CI: 1.01–1.15; *p* = 0.016), urinary manganese (aHR = 1.51; 95% CI: 1.15–1.99; *p* = 0.003), and urinary cobalt (aHR = 1.30; 95% CI: 1.03–1.64; *p* = 0.030) with the likelihood of death in these patients. Each incremental unit of these elements was linked to an 8%, 51%, and 30% rise in the risk of mortality, respectively. Conversely, we also noted an inverse correlation with tissue copper levels, where each unit increase was associated with a 1% decrease in the risk of death (aHR = 0.99; 95% CI: 0.98–0.99; *p* = 0.045).

**TABLE 5:** Cox regression between mortality and trace element concentrations in prostate tissue (μg/dL) and urine (μg/g creatinine) in 94 prostate cancer patients.

Trace element	aHR	SE	*t* value	*p* value	95% CI
As‐U	0.97	0.04	−0.60	0.550	0.90–1.061
**Co‐U**	**1.30**	**0.16**	**2.17**	**0.030**	**1.03–1.64**
Cr‐U	0.62	0.36	−0.83	0.408	0.20–1.93
**Mn‐U**	**1.51**.	**0.21**	**2.97**	**0.003**	**1.15–1.99**
Pb–U	0.99	0.04	−0.20	0.842	0.92–1.08
Se‐U	1.01	0.02	0.49	0.621	0.97–1.05
Zn‐U	1.00	0.01	−0.43	0.666	0.99–1.01
Cd‐U	0.44	0.36	−1.01	0.313	0.09–2.18
Cu‐U	1.005	0.01	0.85	0.396	0.99–1.02
**Sr‐U**	**1.08**	**0.04**	**2.40**	**0.016**	**1.01–1.15**
Al‐T	0.97	0.02	−1.53	0.127	0.94–1.01
As‐T	0.85	0.16	−0.84	0.400	0.57–1.24
Co‐T	1.01	0.03	0.20	0.841	0.95–1.06
Cr‐T	1.04	0.03	1.41	0.159	0.99–1.09
**Cu‐T**	**0.99**	**0.01**	**−2.00**	**0.045**	**0.98–0.99**
Mn‐T	1.04	0.03	1.29	0.197	0.98–1.10
Ni‐T	0.99	0.03	−0.51	0.61	0.93–1.05
Pb‐T	1.03	0.16	0.23	0.821	0.77–1.39
Se‐T	1.11	0.08	1.47	0.142	0.97–1.26
Sr‐T	7.42	18.95	0.78	0.433	0.05–1110.82
Zn‐T	0.99	0.01	−0.93	0.351	0.97–1.01

*Note*: The bold values presented in Table 5 represent the statistically significant results found in our analysis (with *p* < 0.05).

Abbreviations: 95% CI, 95% confidence interval; HRa, adjusted hazard ratio; SE, standard error; ‐T, prostate tissue; ‐U, urine.

**FIGURE 2:**
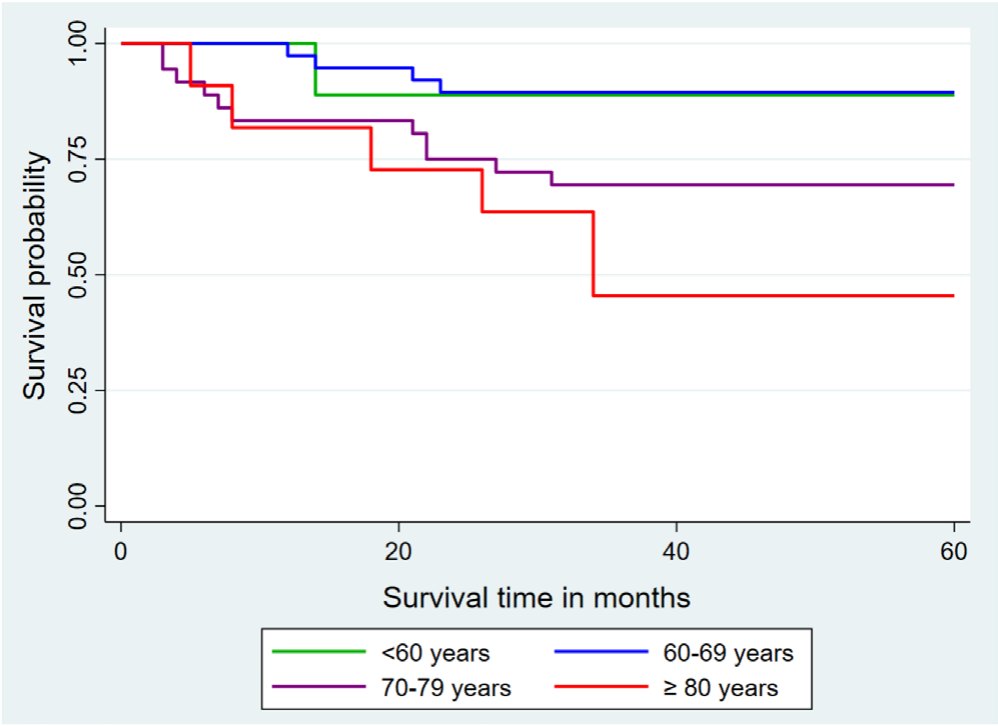
Kaplan–Meier curves of overall survival at 60 months as a function of age.

The risk of death among the patients in this study escalated with age. Older patients had a shorter survival interval compared to younger patients (Figure 2). Furthermore, a statistically significant difference in the 5‐year overall survival rate was noted between various age groups (Log‐rank test = 11.05; *p* = 0.0115).

The 5‐year overall survival rate of patients with a Gleason score > 7 was not significantly different from that of patients with a Gleason score ≤ 7 (Log‐rank test = 0.60; *p* = 0.4376).

**FIGURE 3:**
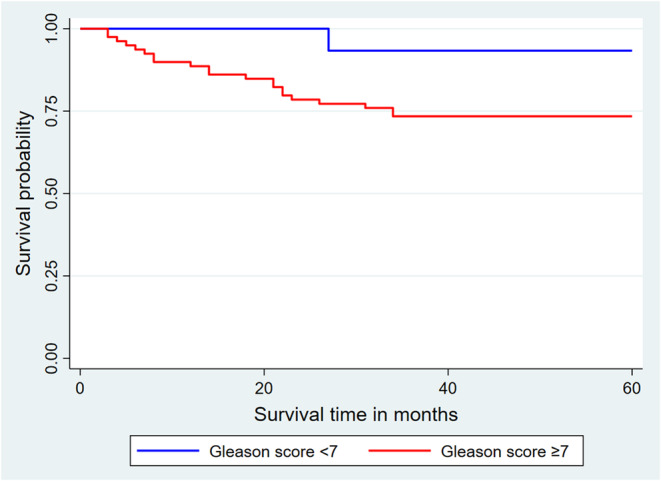
Kaplan–Meier curves for overall survival at 60 months as a function of Gleason score.

The Kaplan–Meier curves for these two Gleason score groups are shown in Figure 3.

**FIGURE 4:**
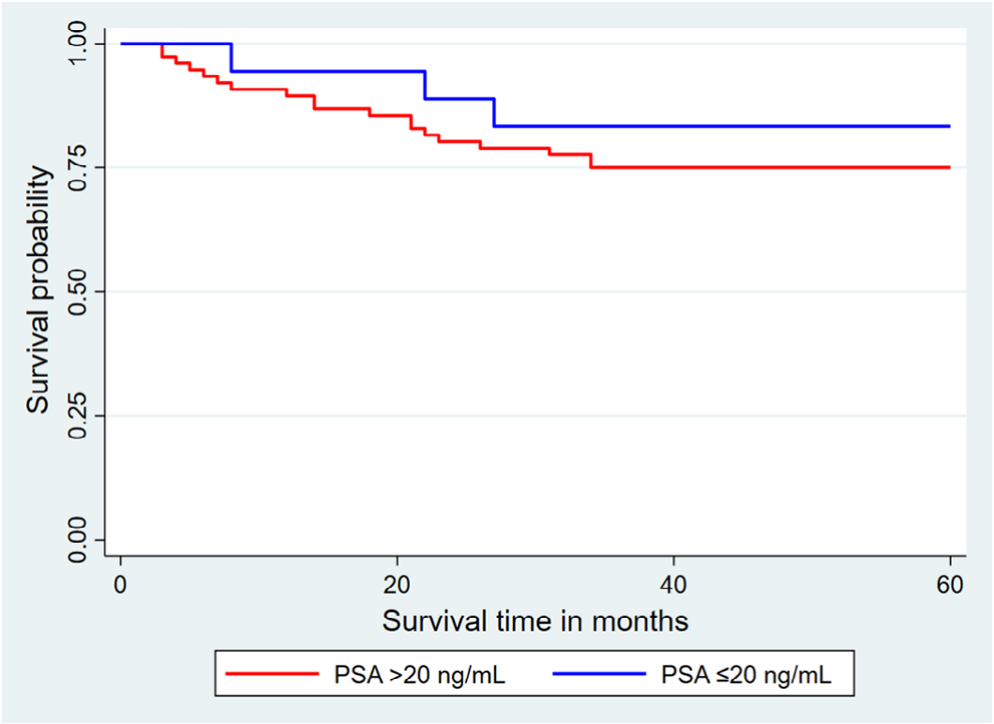
Kaplan–Meier curves of overall survival at 60 months as a function of PSA.

The likelihood of death in patients with PSA levels greater than 20 ng/mL was higher compared to those with PSA levels less than or equal to 20 ng/mL. However, the Log‐rank test revealed no significant variance in the 5‐year overall survival rates between these two PSA groups in PCa patients (Log‐rank test = 2.67; *p* = 0.1023). Figure 4 illustrates the Kaplan–Meier curves depicting the 5‐year overall survival rates among the various PSA levels.

**FIGURE 5:**
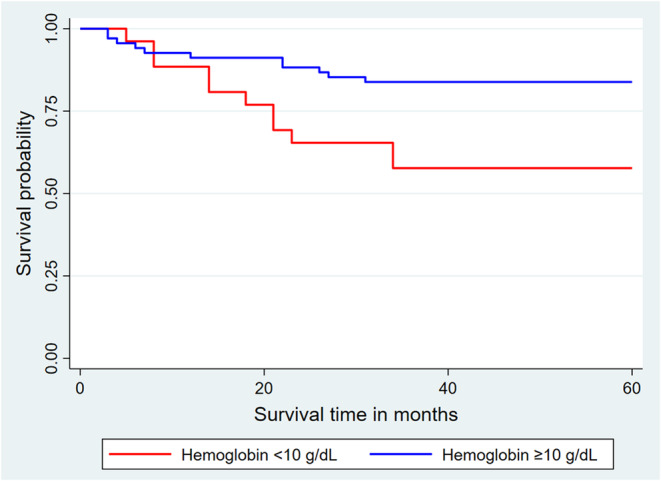
Kaplan–Meier curves of overall survival at 60 months as a function of hemoglobin.

Patients with hemoglobin levels < 10 g/dL were found to have a higher likelihood of mortality compared to those with hemoglobin levels ≥ 10 g/dL (Log‐rank test = 7.02; *p* = 0.0081). Kaplan–Meier curves illustrating the overall survival at 60 months for different hemoglobin levels are presented in Figure 5. During a 20‐month follow‐up post‐diagnosis, patients with hemoglobin levels < 10 g/dL exhibited an overall survival rate of below 75%.

**FIGURE 6:**
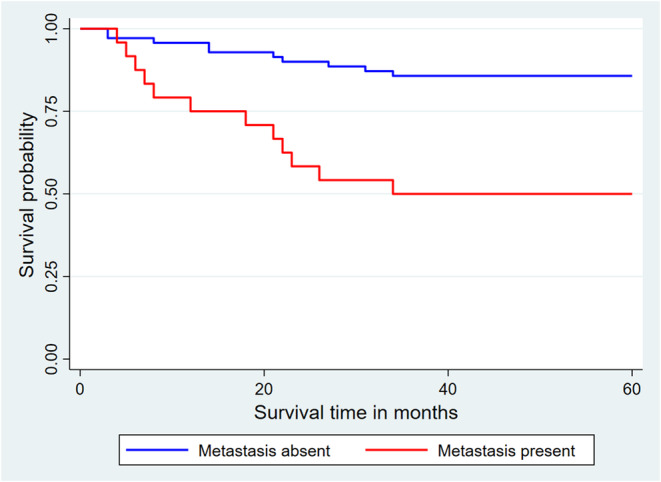
Kaplan–Meier curves for overall survival at 60 months as a function of the presence of metastasis.

The risk of death among patients included in this study increased with the presence of metastases, resulting in a statistically significant difference in 5‐year overall survival between patients with metastases and those without (Log‐rank test = 14.55; *p* < 0.0001). Patients without metastases died at a longer interval than those with metastases (Figure 6).

## 4 Discussion

The study findings in Lubumbashi (in the DRC) highlighted specific factors that impact mortality in PCa patients. Elevated levels of Cobalt, Manganese, and Strontium in urine, along with factors such as advanced age, high PSA levels, low hemoglobin levels, and the presence of metastases, were identified as key determinants of patient mortality. Interestingly, higher prostate tissue copper concentrations were linked to reduced mortality among the patients in this study.

Advanced age is associated with increased mortality in PCa patients due to several factors. Older patients are more likely to suffer from multiple co‐morbidities such as cardiovascular disease or diabetes, which not only complicate PCa treatment but also increase the risk of mortality in terms of competing co‐morbidities [18, 19]. Additionally, PCa in these patients is frequently diagnosed at more advanced stages, often due to less frequent screening and overlooked symptoms [5, 18, 20]. Lastly, treatment options for elderly patients may be restricted due to their reduced tolerance to aggressive treatments like chemotherapy or surgery.

In our study, we found a significant association between elevated PSA levels and mortality in PCa patients. PSA serves as a crucial indicator of PCa progression. Increased levels of PSA in the blood are typically linked to a larger tumor burden and a more aggressive disease advancement, often indicating advanced cancer or biological recurrence following treatment. Moreover, PSA is frequently utilized to track the response to treatment [21, 22].

The results of the present study show that hemoglobin levels were also correlated with mortality in PCa patients. Low hemoglobin levels, or anemia, are common in these patients due to factors like bleeding, inadequate nutrition, or treatment side effects such as hormone therapy and chemotherapy [23, 24]. Anemia can weaken patients, reduce their ability to tolerate treatment, and increase mortality. Additionally, low oxygenation can worsen the patient's overall condition and promote cancer progression, thereby contributing to increased mortality. In a systematic review, Caro et al. [25] found that anemia was associated with shorter survival times for PCa patients.

As our study showed, the presence of metastases was significantly associated with an increased risk of mortality. The presence of metastases indicates an advanced stage of the disease. Metastatic cancer is more challenging to treat and is linked to a poorer prognosis. Metastases can result in complications in various organs, such as the bones, liver, or lungs. These complications can cause pain, fractures, organ failure, and other serious conditions that elevate the risk of mortality [5, 26, 27].

The positive correlation we observed between elevated urinary strontium levels and an increased risk of mortality in PCa patients is particularly intriguing. This finding echoes the complex nature of strontium, as demonstrated in the existing literature.

On the one hand, the tumor‐killing properties of strontium, particularly in the form of the radioisotope strontium‐89, are well documented. Studies such as those by Kuroda [28] and Gunawardana et al. [29] illustrate the therapeutic benefits of strontium‐89 in managing bone metastases associated with PCa, highlighting its potential to relieve pain and potentially prolong survival. On the other hand, our results suggested that natural strontium, when present at high levels in the body, may contribute negatively to patient outcomes. This paradoxical role of strontium calls for further study of the mechanisms by which it interacts with cancer tissues and affects overall mortality.

Manganese levels showed an even stronger association with mortality risk, with a 51% increase noted in the present study. The literature presents a dichotomy in the role of manganese in PCa. The study by Hernroth et al. [30] highlights the ability of manganese to reduce the viability of PCa cells by inducing apoptosis, suggesting a potential therapeutic angle. Conversely, the meta‐analysis by Liwei et al. [31] of manganese superoxide dismutase gene polymorphisms reports no significant association with PCa susceptibility, indicating that the relationship between manganese and PCa may be more nuanced than previously thought. Our results contribute to this complexity by suggesting that if manganese can exert cytotoxic effects on cancer cells in vitro, high urinary levels could be harmful and indicate advanced disease or a disturbed metabolism that adversely affects patient survival.

Elevated urinary cobalt levels were also associated with an increased risk of mortality, with a 30% increase in risk associated with higher cobalt levels. The systematic review of cobalt levels in human prostate [32] suggests that cobalt may play an important role in prostate carcinogenesis and could potentially act as a biomarker for the disease. The finding that higher urinary cobalt levels correlate with increased mortality risk adds another layer of evidence to the hypothesis that cobalt may be involved in the pathogenesis of PCa. This finding aligns with Pietrzak et al. [15], who demonstrated that elevated serum cobalt levels may be indicative of worse outcomes in PCa patients, suggesting that optimal levels of this element may have risk effects. The role of cobalt in cancer progression remains an area of active investigation, and its association with mortality could provide a new avenue for prognostic biomarker development in PCa. The present study supports the idea that monitoring cobalt levels could provide valuable prognostic information and potentially guide therapeutic interventions.

In contrast, we identified an inverse relationship between tissue copper levels and mortality risk, indicating a potential protective effect of copper against PCa mortality. This is in line with the work of Machado et al. [33], who report the development of novel copper complexes in association with selective cytotoxicity against PCa cells, offering a glimmer of hope for targeted cancer therapies. This contrasts with studies like those by Lubiński et al. [16], which found that a copper level in the highest quartile was associated with increased mortality. However, the use of copper in a therapeutic context must be approached with caution, as evidenced by the research of Shokrzadeh et al. [34], who warned of the cytotoxic effects of copper nanoparticles on normal cells. This suggests that while some forms of copper may be beneficial in the treatment of PCa, there is a fine line between therapeutic doses and toxic exposure.

While recent literature has emphasized the potential protective effects of trace elements such as selenium and zinc in PCa survival [16, 17], our findings did not reveal a significant association between these elements and mortality. These findings have important implications for clinical practice and health policy in the DRC. Surveillance of trace elements could provide valuable information for the management of PCa, although further research is needed to confirm these associations and understand the underlying mechanisms. In addition, improving access to diagnosis and treatment, including regular monitoring of PSA levels and care for renal and anemic complications, is crucial to improving patient outcomes.

Furthermore, recent advances in prostate cancer prognosis have highlighted the role of liquid biopsy biomarkers in diagnosis and prognosis, as outlined by Matuszczak et al. [35]. Incorporating biomarkers such as Progensa PCA3, MyProstateScore, ExoDx, SelectMDx, PHI, 4K, Stockholm3, and ConfirmMDx into standard clinical practice has the potential to enhance early detection and personalized treatment strategies. Although our study did not include liquid biopsy markers, future studies could explore these emerging diagnostic tools to improve the precision of prognosis and tailor treatment approaches in PCa patients.

Another significant area of discussion pertains to the treatment of oligometastatic prostate cancer (OMPCa). Oligometastatic disease has emerged as a distinct clinical entity, with evolving therapeutic strategies aimed at improving survival outcomes. Recent studies have shown that metastasis‐directed therapy, including stereotactic radiotherapy and surgical metastasectomy, can enhance survival rates in patients with a limited metastatic burden [36]. While our study focuses primarily on trace element influence and overall clinical factors, integrating metastasis‐directed therapy into future treatment strategies for oligometastatic prostate cancer could significantly impact patient outcomes in regions like Lubumbashi.

This study has several limitations. Firstly, the observational nature of the study limits the ability to establish causal relationships between demographic, clinical, laboratory, therapeutic features, and levels of trace elements and mortality in PCa patients. The observed associations may be influenced by unmeasured confounding factors. In addition, the restricted geographical sampling to the Haut‐Katanga province in the DRC and the limitation of the sample size (*n* = 94) may limit the generalizability of the results to other populations or geographical contexts. Furthermore, although standardized techniques were used to measure trace element levels, variations in sample handling and analysis may introduce measurement errors that affect the accuracy of the results. Finally, despite the adjustments made for several clinically relevant variables, it is possible that other unmeasured confounding factors (such as dietary habits, environmental exposure, and medical history) may have influenced the results. These uncontrolled confounding factors may therefore limit the interpretation of the associations observed.

Despite these limitations, the study has several notable strengths. The longitudinal nature of the study, with a follow‐up over 5 years, enables the long‐term effects of trace element levels on mortality to be observed, providing valuable temporal perspectives on disease progression. In addition, the exhaustive inclusion of PCa patients from several health institutions in Lubumbashi ensures that the study population is highly representative, increasing the robustness of the results. The use of state‐of‐the‐art techniques for the determination of trace elements, such as the OptimaTM 8300 ICP‐OES spectrometer, guarantees accurate and reliable measurements, reinforcing the validity of the associations observed. Furthermore, the application of Cox regression models adjusted for various risk factors enables a rigorous assessment of the relationships between trace element levels and mortality, taking into account potential confounders. Finally, the results of this study provide important perspectives for the clinical management of PCa patients, suggesting that monitoring trace element levels could be integrated into treatment protocols to improve survival outcomes. These clinical and practical contributions enrich our understanding of the roles of trace elements in PCa progression and mortality.

## 5 Conclusion

PCa remains a fatal disease, especially in low‐income countries where diagnosis is often delayed. The study's findings indicated that elevated urinary levels of specific trace elements (cobalt, manganese, and strontium), along with factors like advanced age, anemia, high PSA levels, and metastases, were predictive of mortality in the study cohort. Conversely, a higher concentration of copper in prostate tissue was found to have a protective effect. These results have the potential to inform the development of personalized prevention and treatment approaches for PCa patients, aimed at enhancing survival rates. Nevertheless, further research is essential to confirm these correlations and unravel the underlying mechanisms.

## Author Contributions

P.M.M., P.D.K.D., O.M., W.K.A., and D.M.M. conceptualized the study and designed the methodology; P.M.M., O.M., and G.I.N. developed the software; P.M.M., O.M., and A.M.‐L.P. conducted the validation; O.M., C.L.B., B.M.L., A.T.M., and M.N.L. performed formal analysis; P.M.M., P.D.K.D., and W.K.A. collected the data; P.M.M., O.M., A.M.‐L.P., G.I.N., and C.L.B. prepared the original draft of the manuscript; P.D.K.D., B.M.L., and D.M.M. provided supervision and contributed to the review and editing. All authors reviewed and approved the final version of the manuscript for publication.

## Ethics Statement

Ethical approval for this study was obtained from the Medical Ethics Committee of the University of Lubumbashi (Approval No. UNILU/CEM/015/2020).

## Consent

Written informed consent was obtained from each participant, with an assurance of confidentiality of information and the right to withdraw from the study at any time.

## Conflicts of Interest

The authors declare no conflicts of interest.

## Data Availability

The datasets used and/or analyzed during the current study are available from the corresponding author upon reasonable request.
